# Retraction: Aerosols Transmit Prions to Immunocompetent and Immunodeficient Mice

**DOI:** 10.1371/journal.ppat.1012396

**Published:** 2024-07-18

**Authors:** The PLOS Pathogens Editors, Johannes Haybaeck, Mathias Heikenwalder, Britta Klevenz, Petra Schwarz, Ilan Margalith, Claire Bridel, Kirsten Mertz, Elizabeta Zirdum, Benjamin Petsch, Thomas J. Fuchs, Lothar Stitz, Adriano Aguzzi

After this article [1] was published and subsequently corrected in [2], the corresponding authors informed the journal editors of additional concerns in the published results. Editorial assessment identified issues in Figures 1, 3 and 4. Specifically:

- The top right region of the Fig 1H C57BL/6 HE panel appears similar to the bottom left region of the far left panel in Fig 3C in [1] and [2].
- The top right region of the Fig 1H C57BL/6 GFAP panel appears similar to the bottom left region of the second from right panel in Fig 3C in [1] and [2].
- The right side of the Fig 1H C57BL/6 Iba-1 panel appears similar to the left side of the far right panel in Fig 3C in [1] and [2].
- The Fig 3A *JH*^*-/-*^ HE panel in [1] appears similar to the Fig 4A *C3C4*^*-/-*^ HE panel when rotated 45°.
- The Fig 3A *JH*^*-/-*^ SAF84 panel in [1] appears similar to the Fig 4A *C3C4*^*-/-*^ SAF84 panel.
- The Fig 3A *JH*^*-/-*^ GFAP panel in [1] appears similar to the Fig 4A *C3C4*^*-/-*^ GFAP panel.
- The Fig 3A *JH*^*-/-*^ Iba-1 panel in [1] appears similar to the Fig 4A *C3C4*^*-/-*^ Iba-1 panel.
- The bottom right region of the Fig 3B LTβR-Ig SAF84 panel in [1] and [2] appears similar to the bottom left regions of the *JH*^*-/-*^ SAF84 panel in Fig 3A in [1] and the *C3C4*^*-/-*^ SAF84 panel in Fig 4A when rotated 150°.
- The top right region of the Fig 3B muIgG Iba-1 panel in [1] and [2] appears similar to the bottom left region of the Fig 4B Newborn *tg*a20 Iba-1 panel.

The corresponding author, AA, stated that several images were acquired of each tissue specimen and pictures were incorrectly assigned to genotypes. They also noted that, in addition to the concerns above, the following panels are incorrect:

- The *Tg*a20 GFAP and C57BL/6 SAF84 panels in Fig 1H.
- The LTβR-Ig HE, GFAP and Iba-1 panels, and the muIgG HE, SAF84 and GFAP panels in Fig 3B.
- The second from left panel in Fig 3C.
- The Newborn *tg*a20 HE, SAF84 and GFAP panels in Fig 4B.
- The Newborn *tg*a20 Kaplan Meyer curve in Fig 4B.

The corresponding author, AA, stated that they identified the original paraffin blocks and the original archive description of the individual genotypes for many of the mouse groups and they provided images generated from new cuts from these blocks re-stained for HE, SAF54, Iba-1 and GFAP.

The corresponding authors requested retraction of the article. In light of the extent of concerns and errors listed above that question the reliability of the reported results and conclusions, and in accordance with the request of the corresponding authors, the *PLOS Pathogens* Editors retract this article.

All authors agreed with the retraction. AA and LS stand by the article’s findings. AA and LS apologize for the issues with the published article.

## References

[1] Haybaeck J, Heikenwalder M, Klevenz B, Schwarz P, Margalith I, Bridel C, et al. (2011) Aerosols Transmit Prions to Immunocompetent and Immunodeficient Mice. PLoS Pathog 7(1): e1001257. doi: 10.1371/journal.ppat.1001257 21249178 PMC3020930

[2] Haybaeck J, Heikenwalder M, Klevenz B, Schwarz P, Margalith I, Bridel C, et al. (2016) Correction: Aerosols transmit prions to immunocompetent and immunodeficient mice. PLoS Pathog 12(2): e1005463. doi: 10.1371/journal.ppat.1005463 26872256 PMC4752327

